# Proposed Treatise on Typhoid and Typhus Fever

**Published:** 1848-01

**Authors:** 


*Proposed Treatise on Typhoid and Typhus Fever.*—Dr. Sutton, of Georgetown, Ky., has it in contemplation to publish a historical and practical treatise on these fevers. as they have prevailed in the interior of Kentucky. The Western Journal predicts that the proposed work will prove a valuable addition to our indigenous literature.

